# A global meta-analysis on the ecological drivers of forest restoration success

**DOI:** 10.1038/ncomms11666

**Published:** 2016-05-19

**Authors:** Renato Crouzeilles, Michael Curran, Mariana S. Ferreira, David B. Lindenmayer, Carlos E. V. Grelle, José M. Rey Benayas

**Affiliations:** 1 Laboratory of Vertebrates, Department of Ecology, Universidade Federal do Rio de Janeiro, Rio de Janeiro 68020, Brazil; 2 International Institute for Sustainability, Rio de Janeiro 22460320, Brazil; 3 Rio Conservation and Sustainability Science Centre, Department of Geography and the Environment, Pontifícia Universidade Católica, Rio de Janeiro 22453900, Brazil; 4 Group of Ecological Systems Design, Institute of Environmental Engineering, Swiss Federal Institute of Technology Zürich, Zürich 8093, Switzerland; 5 Fenner School of Environment and Society, The Australian National University, Canberra, Australian Capital Territory 2601, Australia; 6 Department of Life Sciences, Alcala University, Alcalá de Henares E-28005, Spain

## Abstract

Two billion ha have been identified globally for forest restoration. Our meta-analysis encompassing 221 study landscapes worldwide reveals forest restoration enhances biodiversity by 15–84% and vegetation structure by 36–77%, compared with degraded ecosystems. For the first time, we identify the main ecological drivers of forest restoration success (defined as a return to a reference condition, that is, old-growth forest) at both the local and landscape scale. These are as follows: the time elapsed since restoration began, disturbance type and landscape context. The time elapsed since restoration began strongly drives restoration success in secondary forests, but not in selectively logged forests (which are more ecologically similar to reference systems). Landscape restoration will be most successful when previous disturbance is less intensive and habitat is less fragmented in the landscape. Restoration does not result in full recovery of biodiversity and vegetation structure, but can complement old-growth forests if there is sufficient time for ecological succession.

## Abstract (web summary)

Restoration of degraded ecosystems is known to enhance biodiversity and vegetation structure. Using a global meta-analysis, Crouzeilles *et al*. identify the drivers of restoration success in forest ecosystems at both the local and landscape scales.

## Graphical abstract

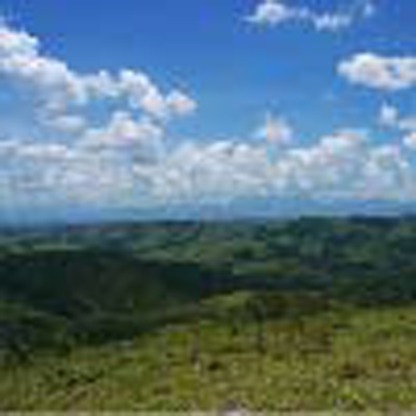

Restoration of previously forested land is a global priority^1^,^2^,^3^. More than two billion ha have been identified globally as potentially suitable for either passive or active forest restoration^4^. International initiatives such as the ‘Bonn Challenge', the first global commitment for forest restoration, seek to restore 150 million ha of disturbed ecosystems by 2020 (ref. 5). Some Latin American and Caribbean countries also have recently launched the ‘Initiative 20 × 20' to restore 20 million ha of forests and other ecosystems by 2020 (ref. 6). This initiative is likely to result in substantial socioeconomic and environmental benefits supported by up to US$ 365 million from private and public funds^6^.

Restoration initiatives are becoming increasingly widespread in many countries, habitat types and a wide range of areas characterized by different socioeconomic and ecological attributes^7^,^8^. Billions of dollars have been spent on ecological restoration methods, technology and knowledge capacity, to achieve effective restoration outcomes^1^,^9^. Nonetheless, these outcomes differ widely among projects, ranging from near-total success to complete failure^10^. The ecological drivers (or factors) underpinning the success of ecological restoration (for example, refs 11, 12, 13), defined as a return to a condition used as a reference (hereafter termed ‘restoration success'), remain unclear. Yet, knowledge of such drivers is urgently required to guide more cost-effective restoration actions on the ground^14^,^15^. Therefore, we posed two key questions: (i) what are the main ecological drivers of forest restoration success for biodiversity (measured through ecological metrics such as abundance, richness, diversity or similarity) and structural features of vegetation (hereafter termed ‘vegetation structure') at both the local (from <1 to 160 ha; based on the data of ref. 11) and landscape (from 7,854 to 6,283,200 ha) scale? And (ii) does restoration success change across different geographic regions and ecological metrics used to assess biodiversity?

To address these two key questions, we conducted a global meta-analysis of the most comprehensive data set gathered to date on forest restoration success. It encompassed 269 primary studies, 221 study landscapes (based on the geographic coordinates reported by the primary studies), 53 countries and six geographic regions, and contained 4,645 quantitative comparisons between reference systems and either restored or degraded systems (Fig. 1). Our extensive analysis included all recorded studies compiled by seven key reviews on either biodiversity recovery or succession in restored and/or degraded systems. We selected studies that met the following three criteria: (i) were conducted in forest ecosystems, (ii) had multiple sampling sites to measure biodiversity and/or vegetation structure in reference and restored and/or degraded systems (that is, replicates for all systems) and (iii) used old-growth or less-disturbed forests as a reference (benchmark) for the system under study.

We defined reference systems as old-growth or less-disturbed forests based on the definition presented in primary studies (as adopted by refs 9, 11, 16), restored systems as selectively logged forests or forests in their initial or secondary stage of succession (that is, areas that regenerated after complete or partial clearance) and degraded systems as a result of different types of human land use (plantation or agriculture). The degraded systems represent the initial degradation level—that is, often the starting point of the restoration process^9^. We classified biodiversity into five broad taxonomic groups (mammals, birds, herpetofauna, invertebrates and plants) and vegetation structure into five measures related to ecological succession (density, litter, cover, biomass and height).

Here we reveal the main ecological drivers of forest restoration success, at both the local and landscape scale: the time elapsed since restoration began, disturbance type and landscape context. Restoration does not result in full recovery of biodiversity and vegetation structure, but forest landscape restoration will be most successful when: (i) there is sufficient time for ecological succession, (ii) previous local disturbance is of low intensity and (iii) habitat is less fragmented in the landscape context. Although forest restoration is no substitute for the protection of old-growth forests, its complementary value can help to reduce rates of biodiversity and vegetation structure loss, offering unrestricted support for continued investment in landscape restoration initiatives.

## Results

### Restoration enhances biodiversity and vegetation structure

We used the response ratio to quantify the standardized effect size^17^ of restoration success between restored or degraded and reference systems within the same study (for example, refs 11, 18). Values for measures of biodiversity and vegetation structure were lower in restored than in reference systems (median effect size ranged from −0.1 to −0.26 and from −0.16 to −0.42, respectively) (Fig. 2a), but higher in restored systems (−0.1 to −0.42) than in degraded systems (−0.23 to −1.2) (Fig. 2b). Differences between reference and restored or degraded systems were lower for biodiversity (<−0.62) than for vegetation structure (<−1.21) and varied among taxonomic groups and measures of vegetation structure (Fig. 2a,b). Median values of effect size were 15–84% and 36–77% higher in restored than in degraded systems for measures of biodiversity and vegetation structure, respectively, and 10–26% and 16–42% lower in restored than in reference systems, respectively (Supplementary Table 1).

### Main ecological drivers of forest restoration success

We fitted generalized linear models to compare multiple candidate models that may predict restoration success including different potential ecological drivers at both the local and/or landscape scale. We identified three drivers of restoration success when comparing reference versus restored systems for biodiversity and vegetation structure (Table 1). These drivers varied in magnitude of impacts among taxonomic groups and measures of vegetation structure (Table 1). At the local scale, the drivers were: (i) time elapsed since restoration began (for plants, cover and biomass) and, (ii) disturbance type (for invertebrate, plants, density and biomass). At the landscape scale, the driver was: (iii) the largest forest patch (for litter). In addition, restoration success was not influenced by geographic region, but it changed across ecological metrics for birds. The null model was the most frequently selected model for mammals, herpetofauna and height. The absolute response ratios were always negatively influenced by time and the largest patch size, that is, the converse for the interpretation of restoration success (Supplementary Table 2). For birds, the difference between restored and reference systems was significantly higher for abundance than for all other ecological metrics, such as richness, diversity and similarity (Supplementary Table 2). For invertebrates, plants, density and biomass, the difference between restored and reference systems were significantly higher for secondary forest than for selectively logged forest (Supplementary Table 2).

## Discussion

A previous global meta-analysis that comprised different terrestrial and aquatic ecosystems to those presented here indicated that restoration of degraded systems enhanced overall biodiversity by 44% (ref. 9). Our larger data set focused only on forest ecosystems and it revealed that enhancement of overall biodiversity also differs among taxonomic groups. Nevertheless, the recovery of vegetation structure (for example, see ref. 19) and species similarity and composition^11^,^20^ are likely to take orders of magnitude longer than species abundance and richness, which represent 73% of our biodiversity data set. Thus, the outcome of restoration success will depend on which ecological attribute is considered.

Although several factors can influence the outcomes of restoration success^16^,^18^,^20^,^21^,^22^, for the first time, we have identified the main ecological drivers of forest restoration success for invertebrates, plants and a broad range of measures of vegetation structure (density, cover and biomass; Table 1)—the time elapsed since restoration began and/or the disturbance type. Hence, time is required for restored systems to reach similar values of those found in reference systems. Time has been shown as a key factor contributing to ecosystem restoration^11^,^20^,^23^,^24^ and we found similar results for plants, cover and biomass. For biodiversity (except plants), our data set mostly comprises ecological metrics of abundance and richness, which take far shorter to recover than similarity and composition^11^,^20^. This probably prevented us from detecting a strong influence of time on the diversity of taxonomic groups other than plants. Thus, it is likely to be that plant biodiversity mediates the long-term recovery of vegetation structure through ecological succession (for example, see refs 25, 26, 27). For instance, long periods are required for the forest canopy to develop from an open state dominated by pioneer species to a closed state dominated by late successional species in old-growth forests^14^, but a fully closed canopy may not be necessary for birds (excluding habitat specialists) that can make use of immature forest^28^. For this taxonomic group, populations in restored systems were not as abundant as in reference systems (Supplementary Table 2). Thus, for birds, which typically have a high number of individuals, abundance appears to represent a conservative and meaningful ecological metric to effectively assess restoration success.

Disturbance type was another key driver of restoration success (Table 1). We found that selectively logged forests were more similar in biodiversity and vegetation structure to reference systems than secondary forests that suffered higher intensity disturbances. Several arguments have been proposed to explain this pattern, such as proximity to reference systems that will help reverse extinction debts^16^. We included different metrics of landscape context in our analysis, but our results revealed that selectively logged forests are ecologically more similar to reference systems than secondary forests. As most of the landscapes in our study were located in the Neotropics (38%), where a large amount of forest area results from passive recovery following agricultural abandonment^14^,^21^,^29^, one would expect to find more depleted communities and simplified measures of vegetation structure compared with other forest ecosystems. A recent global meta-analysis^16^ suggested that restoration actions should be focused on selectively logged forests, which would offer the greatest conservation benefits for biodiversity. However, our results suggest that restoration actions should target areas with an intermediate degree of disturbance (degraded/secondary forests), as the gains for biodiversity and vegetation structure offered by selectively logged forests are lower, because they are ecologically more similar to reference systems. As suggested by refs 30, 31, areas subject to an intermediate degree of disturbance may also provide the greatest increase in conservation value per unit cost.

Drivers operating at the landscape scale are also known to influence restoration success^11^,^22^,^32^. Our results suggest the landscape context affects the recovery of litter accumulation. An increase in the largest patch size in landscapes enhanced litter accumulation (Table 1), although it may not been driven exclusively by this landscape variable. In our data set, forest cover, mean, largest size and isolation of forest patches in the landscape were strongly and positively correlated. That is, less fragmented landscapes had higher amounts of forest cover along with larger forest patches than more fragmented landscapes, which supported smaller forest patches. Thus, our results suggest the landscape context affects the recovery of similar amounts of litter accumulation as in reference systems. The previous global meta-analysis of ref. 32, which used a similar data set to this study, revealed a clear pattern of increasing restoration success and decreasing uncertainty across the gradient of contiguous forest cover for biodiversity and vegetation structure. In our study, we did not detect an over-riding effect of forest cover on restoration success in relation to a broader set of potential drivers. Thus, although forest cover can affect restoration success for biodiversity and vegetation structure, it is not the main driver of restoration success.

Our results, mainly the overall lack of effects at the landscape scale, are highly dependent on the coarse spatial resolution of the land cover data (1 km resolution) and on the average values of biodiversity and vegetation structure across sites within a single system (restored or reference) in a single study. This latter procedure provided estimates of uncertainty, but meant our data points related to clusters of sites rather than precise localities. Finer resolution land cover data are required to better assess the effects of forest cover and landscape configuration on restoration success. Perhaps unsurprisingly, we could not identify the main drivers of restoration success for forest mammals, herpetofauna and height. Mammals, for example, may increase in species richness and abundance in disturbed systems as a consequence of colonization by non-native and generalist species^16^,^22^. Thus, our results reflect the pattern produced by analysing all species with no distinction among forest specialist and generalists, and different ecological metrics used to assess biodiversity (which can affect restoration success; Table 1). According to ref. 16, the difference between degraded and restored systems, on one side, and reference systems, on the other side, was higher when the analysis was restricted to forest specialist species. Future systematic reviews should focus on finer resolution landscape data, the response of forest specialist species and more sensitive ecological metrics of community change such as similarity indices. This will help improve our understanding of the driving forces behind ecological restoration, in particular for those taxonomic groups and measures of vegetation structure that could not be addressed in our analysis.

For the first time, we identified the time elapsed since restoration began and/or the disturbance type as the main ecological drivers underpinning forest restoration success, at least for invertebrates and plant biodiversity and most measures of vegetation structure. Previous research has shown that time is a key factor for explain restoration success of biodiversity and vegetation structure in forests (for example, refs 11, 20, 23, 24). Nonetheless, these studies often analysed time only, ignoring other potential drivers and thus potentially failing to identify the most important drivers of success. Our global meta-analysis has considered a range of potential drivers at both the local and landscape scale, and included data spanning many habitat types, restoration ages, habitat configurations and socio-economic contexts. As restoration success was not directly influenced by geographic region (Table 1), we encourage the exchange of empirical experience and knowledge in restoration initiatives across jurisdictions.

The time elapsed since restoration began strongly drives restoration success in secondary forests, but not in selectively logged forests, which are ecologically more similar to reference systems. Another key driver is landscape context. Thus, forest landscape restoration will be most successful when previous local disturbance is less intensive and habitat is less fragmented in the landscape context. Stakeholders should reconsider spending resources on restoring selectively logged forests, as such resources are better spent on restore areas with an intermediate degree of disturbance. Restoration does not result in similar values (that is, full recovery) of biodiversity and vegetation structure to those of old-growth forests; thus, primary forests are indeed irreplaceable for the maintenance of biodiversity^16^. However, over longer periods of ecological succession, the complementary value of restored forests to existing primary forests will increase, thus providing a strong rationale for continued investment in landscape restoration initiatives.

## Methods

### Data collection

Our literature search was performed on all quantitative studies compiled by seven key reviews of scientific literature that included quantitative data of biodiversity recovery or forest succession in restored and/or degraded forest ecosystems^9^,^11^,^16^,^20^,^21^,^33^,^34^. The review of ref. 11 also included data from previous reviews on secondary forest growth and ecological restoration (for example, see refs 12, 29, 35, 36, 37, 38, 39). Despite the common focus, many of the compiled studies in each of the seven reviews were different, thus encompassing different search and inclusion criteria (Supplementary Table 3). Thus, we focused on screening the compiled studies by these reviews that used different inclusion criteria rather than performing a new literature search. We screened the reference list of these reviews for studies that followed the three criteria defined in the main text. After we looked at the title and abstract of all studies, the potential study candidates for inclusion were assessed in detail. As our main interest was to represent restoration outcomes for multiple species, community-level biodiversity data were gathered for ecological metrics that assess multiple species (richness, diversity and similarity) or population patterns in a community (abundance). This resulted in the most comprehensive data set gathered to date on forest restoration success. This comprised 269 selected studies after excluding those that were recorded in more than one review and the additional studies that were suggested by specialists (see Supplementary Information).

Some potential methodological pitfalls often pertain to comparisons of biodiversity and vegetation structure between restored and reference systems (for example, see refs 12, 27). For example, reference systems often: (i) occur in different ecological zones or soil conditions than restored systems, (ii) are assessed in a unique site (no replication), (iii) are larger than restored areas and (iv) suffer less edge effect than restored systems, which can confound effects of the time elapsed since restoration began. These potential pitfalls had a low influence in our study because: (i) many of our sampling sites were <5 km apart; thus, reference and restored systems were more likely to be in the same ecological zone; (ii) we included only studies with replicates for both systems; and (iii) we assessed the influence of landscape variables, such as patch size and edge effect.

### Data extraction

We recorded for each study the following information: (i) study year, (ii) country, (iii) geographic region, (iv) latitude and longitude, (v) disturbance type, (vi) the time elapsed since restoration began, (vii) restoration activity, (viii) ecological metric used to assess biodiversity and (ix) quantitative measure of biodiversity response and/or vegetation structure for reference and restored and/or degraded systems. These were the most common data available in the selected studies. We gathered data for six of the seven biogeographic realms proposed by Olson *et al*.^40^, which were used to represent different geographic regions (Antarctic was not represented). Disturbance type was divided into secondary forest and selectively logged forest according to ref. 16. Secondary forests are areas that were completely or partially cleared and then regenerated (either passively or actively) after disturbance ceased. Thus, secondary forests include selectively logged forests, but selective logging was considered as a different disturbance type following the work by Gibson *et al*.^16^, which suggests that these forests are largely affected by a single cutting cycle, whereas secondary forests can be affected by many cutting cycles, fire and/or other types of disturbance. It is important to highlight that our classification was based on the definition of the primary study. The time elapsed since restoration began was measured in years (for example, see refs 11, 20, 23, 24), that is, a higher value means more time a forest has been restored. Restoration activity was divided into passive regeneration, active management or planting. Passive restoration is forest re-growth following land abandonment or the cessation of disturbance pressure (for example, exclusion of grazing), active management represents manipulating disturbance regimes through the use of thinning and burning, and active planting represents plantation of tree species to influence the successional trajectory of recovery (for example, see refs 41, 42). Ecological metrics were classified as abundance (for example, number, proportion, frequency and density of individuals, equitability, capture rates, captures per effort time), richness (for example, observed, estimated, rarefied, genera, family and guild richness, and species density), diversity (for example, Shannon index, Simpson index, Margalef index, Fisher alpha and evenness), species similarity (for example, Sorenson index, Morisita–Horn index, analysis of similarities (ANOSIM), principal component analysis (PCA), multidimensional scaling (MDS), Mantel, Jaccard index, Bray Curtis and Euclidean distances) or others (for example, colonization, extinction, visitor, encounter and removal seed rates, fruit production, recruitment and proportion of traits per state). Following ref. 3, we also included ecological metrics of the ‘others' class in our analysis, as we can expect restored systems to have higher extinction rates than reference systems. We classified biodiversity into five broad taxonomic groups (mammals, birds, herpetofauna, invertebrates and plants) and vegetation structure into five measures related to ecological succession (density, litter, cover, biomass and height). Density refers to the number of individuals per unit area, litter is the amount of leaf litter on the substrate, cover is the area covered by vegetation (measured in three strata—floor, understory and canopy), biomass is the amount of below- and/or above-ground biomass produced (for example, basal area) and height is vegetation height above the ground.

Few studies had information at the landscape scale, thus we mapped forest areas within each study landscape. We estimated five widely used metrics of habitat loss and fragmentation^43^ within each study landscape: (i) percentage of forest cover, (ii) mean size of all forest patches, (iii) size of largest forest patch, (iv) isolation of forest patches (measured as mean nearest-neighbour distance among patches) and (v) edge:area ratio of forest patches. Edge:area ratio was measured as the edge to area ratio of all forest patches. These metrics were consistent with those available in FRAGSTATS, a specific software to calculate landscape metrics^44^. We constrained landscape size based on a previous global meta-analysis that was conducted to identify the scale of effect (that is, best landscape size) of forest cover on restoration success for biodiversity and vegetation structure to avoid arbitrary decisions in this respect^32^. The most plausible scale of effect was used to delimit landscape size: 10 km radius for plants, 5 km radius for mammals, invertebrates, cover and height, and 100 km radius for litter (but this had a small sample site, resulting in poorly fitted models that cannot represent the scale of effect). For density, biomass, birds and herpetofauna, the null model (only intercept and error as parameters) was considered among the plausible models. Thus, for these we used the most frequent landscape size found for biodiversity and vegetation structure (5 km radius). The results in ref. 32 represent large landscape sizes (ranging from 7,854 to 6,283,200 ha), but it is important to note that restoration success (measured in a similar way as in this study) was calculated for an average of sampling sites (reference and restored systems) per quantitative comparison; thus, the ‘study landscape' was used as the unit of analysis rather than sampling site (similar as we did for this study).

To map forest area and estimate the five landscape metrics, we used a recently developed 1 km resolution consensus land cover data set^45^. The data set is presented on both ‘full' (combining four existing land cover products: DISCover1995, GLC 2000, MODIS 2005 and ESA GlobCover 2008) and ‘reduced' forms (the latter three products). As most of our data were from the Neotropic (77 study landscapes) and Indo-Malay realms (43 study landscapes), which have exhibited a large amount of forest cover change between 1990 and 1995, we chose the ‘reduced' form, to reduce the influence of forest cover change^45^. The data expresses sub-pixel coverage of 12 land cover types, including four potential forest classes. We included only three of the four forest land cover classes (‘Evergreen/Deciduous needleleaf', ‘Evergreen broadleaf' and ‘Evergreen needleleaf'), omitting the ‘Mixed/other' class, because it contains both forest and non-forest land cover types. We summed up total pixel coverage of the three classes and retained only pixels with >60% forest cover. These pixels represent contiguous forest areas that have stronger effects on restoration success than the overall forest cover^32^. Thus, our estimate of source forest cover was conservative and representative of dense, closed-canopy forest areas. Although higher-resolution forest-cover data sets are available, we choose the consensus data set of ref. 45 for the following reasons. First, we judged the consensus product to be robust, as it was based on agreement between products at different times, including two sub-pixel (that is, <1 km) products, thus better capturing land cover classes with a permanent cover (for example, natural forests rather than periodically harvested plantations or changing land use classes). Second, the consensus product has been tested for accuracy in an ecological predictive modelling context for species distribution models^45^. Third, as a single cover estimate, it probably reflects a more accurate picture of average forest cover over the time period of studies that we considered rather than a snapshot of forest cover in the middle of the time span of studies. Finally, rather than focusing on either sampling sites or single sites requiring high-resolution data, our focus was on the landscape scale; thus, a 1-km resolution map with sub-pixel information was sufficient to identify landscape patterns.

To characterize the study site in each landscape, we tried to localize geographic coordinates for each study to lie in the centre of the cluster of sampling sites, so that the ‘study landscape' was used as the unit of analysis rather than the sampling site. When geographic coordinates were not available in the selected studies, we contacted the corresponding authors by email to request this information. The forest cover data ranged from 0 to 100% across study landscapes. All area calculations were conducted with the equal-area World Mollweide projection (EPSG: 54009). All geographic analyses were conducted using the open source Geographic Resource Analysis Support System GIS, vers. 6.4.3 (ref. 46) and Quantum GIS, vers. 2.6.1 (ref. 47).

### Response ratio calculations

The standardized mean effect size is a useful measure to compare two natural groups with respect to some quantitative and normally distributed dependent variable (for example, ref. 48). Nearly half of all published meta-analyses in ecology have used response ratio^49^,^50^. The advantage of response ratio compared with other metrics is that it simply needs a raw mean of the dependent variable for two groups, whereas other metrics also need the variance (or standard deviations (s.d.)) and sample sizes for two groups^51^. Thus, we used the response ratio as the standardized mean effect size, because we were interested in obtaining as much information as possible from the available studies to perform separate analyses for each taxonomic group and measure of vegetation structure. This would not have been possible using a weighted response ratio, as many studies did not provide information on variance or s.d.

For each comparison of either biodiversity response or measure of vegetation structure within the same assessment, we calculated a response ratio between reference and restored or degraded systems. Response ratio was calculated as ln(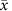 degraded or restored/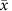 reference), where 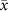 is the mean value for a quantified variable across all sampling sites (that is, extracted from the replicates)^17^. For similarity data, we used the mean difference between restored or degraded and reference systems divided by the mean difference in the reference system; see ref. 11 for further explanation. Negative effect size means that the measured value of biodiversity or vegetation structure in the restored/degraded system was lower than in the reference system, that is, there was a negative impact on the measured value, whereas the opposite holds for a positive effect size. Values around zero are the desired outcome of restoration, that is, restored/degraded systems have reached a benchmark state.

Following refs 9, 16, we inverted the sign of data that are a priori expected to exhibit positive response ratios, that is, higher values in the restored or degraded than in the reference systems. The sign of the following measures was inverted: (i) openness, (ii) introduced species, (iii) grasses, (iv) exotic species, (v) herbs, (vi) open-habitat species, (vii) gap species, (viii) trees of diameter at breast height <10 cm and (ix) bare ground percentage. The study of Meli *et al*.^18^ showed no difference in the meta-analysis results, either when considering or ignoring the inverted response ratios. Thus, we choose to use as much data as possible. Response ratios cannot be calculated for a quantified variable with a zero value; thus, we excluded 88 comparisons that had zero values for restored, degraded or reference systems. This gave us a total of 4,557 quantitative comparisons between reference and restored or degraded systems.

### Meta-analysis

We compared data sets of reference with restored or degraded systems for each taxonomic group and measure of vegetation structure. Each study landscape may have multiple response ratios if they were the focus of multiple studies or if the same study analysed the following: (i) multiple ecological metrics (abundance, richness, diversity and/or similarity); (ii) more than one guild (for example, frugivores and insectivores birds); (iii) lower taxonomic group divisions than applied in our analysis (for example, hemiptera and hymenoptera); or (iv) different years or seasons separately. Thus, to avoid spatial pseudo-replication, we resampled any given data set with replacement (10,000 bootstraps) and used only one comparison per study landscape to generate the median effect size and 95% confident intervals^23^. Outliers were removed to achieve normally distributed residuals, which were checked by plotting residuals^52^.

The percentage enhancement of biodiversity and vegetation structure in restored versus degraded systems was inferred based on the difference between the median values of response ratios for both systems, which were originally compared with reference systems (Supplementary Table 1). We did not compare directly restored versus degraded systems, because our criteria included only studies that compared reference with restored and/or degraded systems. If we had compared restored and degraded systems, it means that some studies could not be inserted in our review, for example, those that compared restored and degraded systems, but did not include reference systems. In addition, our analysis is related to restoration success, that is, return to a reference condition; thus, we were always focused on the use of a reference system as a benchmark to present our main messages.

Meta-analyses may suffer from publication bias, which is the probability that significant results are more likely to be published than nonsignificant results^51^. To test this, both study sample size and associated variance (or s.d.) is required. As stated above, we selected some studies that did not report variance values; hence, it was not possible to evaluate publication bias. However, we believe it is not likely to be a problem in our data set, as there are many studies reporting unsuccessful restoration outcomes^10^ and ∼45% of our data came from a review^16^ that tested and found a low influence of publication bias.

### Potential ecological drivers of forest restoration success

**Figure 1:**
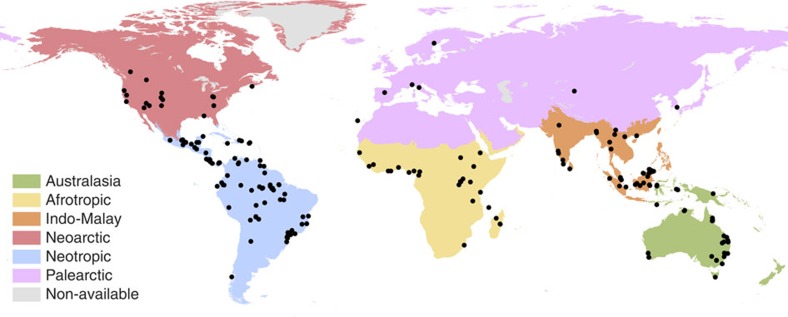
Map of study landscapes (*n*=221) by geographic region. We used the geographic regions as defined by ref. 40. Study landscapes are represented by black dots. Eleven study landscapes are not represented because of a lack of information on their location.

**Figure 2:**
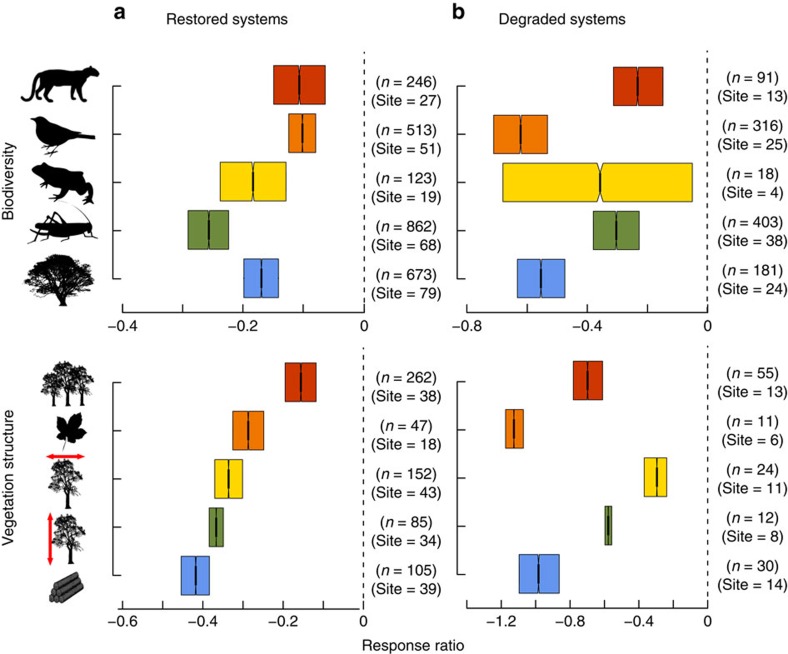
Bootstrapped response ratios. Bootstrapped response ratios for mammals, birds, herpetofauna, invertebrates, plants, density, litter, cover, height and biomass for restored (**a**) and degraded (**b**) systems compared with reference systems. Dashed lines indicate no difference to reference systems. Positive effect sizes indicate higher values of biodiversity or vegetation structure in the restored/degraded systems than in the reference system. The opposite holds for a negative value. Measures of biodiversity and vegetation structure were lower in restored than in reference systems, but higher in restored than in degraded systems. *n*=sample size, site=number of study landscapes (bootstrap sample size used to avoid spatial pseudo-replication). Box plot shows the median value, first and third quartile ranges of resampled response ratios. Notches in boxes (barely visible due to 10,000 bootstraps) represent 95% confidence intervals and non-overlapping notches between boxes imply a significant difference^57^.

We evaluated a total of eight potential drivers of restoration success at both local and landscape scale. The three drivers of restoration success quantified for restored systems at the local scale were as follows: (i) disturbance type according to ref. 16 (namely secondary forest and selectively logged forest), (ii) time elapsed since restoration began and (iii) restoration activity (passive regeneration, active management and planting). The five drivers of restoration success quantified for all forest patches at the landscape scale were as follows: (iv) percentage of forest cover, (v) mean size of forest patches, (vi) size of largest forest patch, (vii) isolation of forest patches (measured as mean nearest-neighbour distance among patches) and (viii) edge:area ratio of forest patches. In addition, we evaluated two variables potentially influencing restoration success, namely (ix) the geographic region, represented by six biogeographic realms^40^ (Fig. 1) and (x) the ecological metric used to assess biodiversity.

### Information-theoretic approach

To identify the main ecological drivers of restoration success for biodiversity and vegetation structure variables, we used an information-theoretic approach^53^. We compared data sets from reference with restored systems for each taxonomic group and each measure of vegetation structure. Thus, our models included a set of ten potential explanatory variables (eight drivers+geographic region+ecological metric) for each taxonomic group and for each measure of vegetation structure, ten separate analyses in total. We additively combined all possible subsets of these ten predictor variables and a null model containing only the intercept and error as parameters. We excluded data points from our data set that did not report all potential drivers or variables potentially affecting restoration success before the analyses, so that all models had the same sample size. Disturbance type, restoration activity, geographic region and ecological metric were categorical predictors with different number of factors; we removed this predictor variable if they did not have at least two factors for a particular data set. Mean size of forest patches and size of largest forest patch were log_10_ transformed. We excluded models that had at least two correlated predictor variables (Spearman's *R*^2^ always >0.7). We used absolute response ratio as the dependent variable, which is easier to be interpreted in the light of restoration success, because we discuss the raw difference between either restored or disturbed and reference systems rather than whether that difference was positive or negative. The data ranged from zero to infinite; thus, absolute response ratio was modelled assuming a Gamma distribution with an identity link function and outliers removed (similar as for meta-analysis) to achieve a normal distribution of residuals^54^.

We calculated for each model the Akaike Information Criterion corrected for small samples (AICc) and the Akaike weight, which indicates the probability that the model_i_ is the best explanatory model within the set of models^53^. The model with lowest AICc, that is, the top-ranked model, is the most plausible to explain a substantial proportion of variance in the data. We also controlled for pseudo-replication as in the meta-analysis (that is, through resampling study landscapes). We calculated the percentage of times that a model was top-ranked after 10,000 bootstraps (*π*_i_) on the basis of AICc^53^. We also used the adjusted *R*^2^ as a coefficient of determination that represents the proportion of the variance explained by the model (that is, the good-of-fitness of the model). The adjusted *R*^2^ is a modified version of generalized *R*^2^ of ref. 55. All analyses were carried out with R 2.12 (ref. 56).

### Data availability

Data, R scripts and the reference list for the 269 studies selected are provided in the Dryad Digital Repository, doi: 10.5061/dryad.k3479.

## Additional information

**How to cite this article:** Crouzeilles, R. *et al*. A global meta-analysis on the ecological drivers of forest restoration success. *Nat. Commun.* 7:11666 doi: 10.1038/ncomms11666 (2016).

## Supplementary Material

Supplementary Information Supplementary Tables 1-3.

## Figures and Tables

**Table 1:** Reference versus restored systems.

Model	*π*_i_	*K*	*w*_i_	*R*^*2*^	sl	*n*
*Mammals*						
Null	45.39	2	0.19	—	19	230
						
*Birds*						
Metric	19.05	6	0.11	0.26	41	394
						
*Herpetofauna*						
Null	57.41	2	0.28	—	15	100
						
*Invertebrates*						
Disturbance type (local)	13.43	4	0.1	0.13	45	626
						
*Plants*						
Time (local)+disturbance type (local)	19.25	5	0.14	0.28	61	519
						
*Density*						
Disturbance type (local)	44.85	4	0.18	0.57	30	237
						
*Litter*						
Largest patch size (landscape)	39.23	3	0.22	0.39	15	39
						
*Cover*						
Time (local)	41.11	3	0.17	0.25	30	82
						
*Height*						
Null	60.61	2	0.18	—	22	38
						
*Biomass*						
Time (local)+disturbance type (local)	34.52	5	0.18	0.41	30	87

*π*_i_, percentage of times a model was top-ranked after 10,000 bootstraps; *k*, number of estimated parameters; Metric, ecological metric; *n*, total sample size; Null, model containing only the intercept and error as parameters; *R*^2^, adjusted *R*^2^; sl, number of study landscapes (bootstrap sample size used to avoid spatial pseudo-replication); Time, time elapsed since restoration; *w*_i_, Akaike weight.

Top-ranked models predicting absolute response ratios (the converse for the interpretation of restoration success) for measures of biodiversity in each taxonomic group (mammals, birds, herpetofauna, invertebrates and plants) and of vegetation structure (density, litter, cover, height and biomass). Significant effects at the local and/or landscape scale are shown in parentheses.
