# Supplementary material for: A global meta-analysis on the ecological drivers of forest restoration success
Source: Nat Commun. 2016 May 19;7:11666. doi: 10.1038/ncomms11666 (PMC4874030; doi:10.1038/ncomms11666)
Supplement: Supplementary Information — Supplementary Tables 1-3. [file ncomms11666-s1.pdf]

Supplementary Table 1 | Median values of response ratios for degraded and restored systems compared with reference systems, and percentage of enhancement of biodiversity and vegetation structure in restored with respect to degraded systems.

	Degraded	Restored	Enhancement (%)
BIODIVERSITY			
Mammals	-0.23	-0.11	52.70
Birds	-0.62	-0.10	83.52
Herpetofauna	-0.35	-0.18	47.49
Invertebrates	-0.30	-0.26	15.16
Plants	-0.55	-0.17	69.23
VEGETATION STRUCTURE			
Density	-0.70	-0.16	77.24
Litter	-1.21	-0.29	76.21
Cover	-0.31	-0.34	*
Height	-0.58	-0.37	36.36
Biomass	-0.98	-0.42	57.14

* no enhancement

Supplementary Table 2 | Reference vs. restored systems. Parameter estimates, standard errors and confidence intervals (2.5 and 97.5%) of models predicting absolute response ratio (the converse for the interpretation of restoration success). Results are presented only for top-ranked models different from the null model (Table 1). Analyses were carried out separately for three taxonomic groups (birds, invertebrates, and plants) and four measures of vegetation structure (density, litter, cover, and biomass).

Model	Coefficients		Confidence Interval	
	Estimate	Std. Error	2.5%	97.5%
BIRDS				
Ecological metric				
Abundance	0.8649	0.0575	0.7499	0.9799
Richness	-0.1192	0.0733	-0.2658	0.0274
Diversity	-0.2628	0.0843	-0.4314	-0.0941
Similarity	-0.1862	0.1178	-0.4219	0.0495
INVERTEBRATES				
Disturbance type				
Secondary Forest	1.0477	0.0712	0.9052	1.1902
Selectively logging	-0.3085	0.2040	-0.7166	0.0996
PLANTS				
Time	-0.0026	0.0012	-0.0051	-0.0001
Disturbance type				
Secondary Forest	1.0038	0.0657	0.8723	1.1353
Selectively logging	-0.2806	0.1244	-0.5295	-0.0318
DENSITY				

Disturbance type				
Secondary Forest	0.9750	0.0764	0.8223	1.1278
Selectively logging	-0.2980	0.1196	-0.5373	-0.0587
LITTER				
Largest patch size	-0.2289	0.0884	-0.4057	-0.0522
COVER				
Time	-0.0083	0.0037	-0.0158	-0.0008
BIOMASS				
Time	-0.0041	0.0017	-0.0075	-0.0006

Time = time elapsed since restoration began.

Supplementary Table 3 | The seven key reviews on either biodiversity recovery or ecological succession of vegetation structure in degraded and/or restored ecosystems, and the additional information regarding the literature search for these reviews.

Review	Literature search (search period)	Keywords	Criteria
Dunn 2004	Web of Science (1994-2001) Cambridge Scientific Abstracts (1982-2001)	1) Disturbance<br>2) Secondary Forest<br>3) Succession<br>4) Pasture<br>5) Logging	i) Clearcut logged sites but not selectively logged sites<br>ii) Species richness or species density<br>iii) Responses of focal taxa across a chronosequence of sites of known ages
Ruiz-Jaen and Mitchell Aide 2005	Restoration Ecology (Vols. 1[1]–11[4])	1) All published papers	i) Only using seeding or planting techniques to assist the restoration process
Bowen et al. 2007	Web of Science (1990-2007)	1) Habitat *and* land abandon* *or* natural regrowth<br>2) Wildlife *and* land abandon*<br>3) Habitat *and* natural regrowth *or* native regrowth *or* natural regeneration *or* native regeneration	i) Article must be published, peer reviewed and written in English<br>ii) Fauna occurrence, abundance, diversity or other estimates of occupancy in regrowth forest<br>iii) Fauna response to regrowth forests and mature forest or cleared land<br>iv) Prior cropping or grazing land use for at least one category of regrowth forest
Rey Benayas et al. 2009	Web of Science (up to 2008)	1) Ecosystem *or* environment *and* service* *or* function* *and* restor* *or* re-creat* *or* rehabilitat*	i) Quantitative measures of variables relating to provision of ecosystem services<br>ii) Quantitative measures of variables relating to biodiversity<br>iii) Information on restored, reference and degraded systems<br>iv) Data on all three types of ecosystem services

Gibson et al. 2011	Web of Science (1975-2010)<br>BIOSIS (1975-2010)	1) Bird*<br>*or* mammal*<br>*or* reptile*<br>*or* amphibia*<br>*or* arthropod*<br>*or* plants*<br>*or* lepidoptera*<br>*or* hymenoptera*<br>*or* arachnid*<br>*or* coleoptera*<br>*or* diptera*<br>*or* isoptera*<br>*and* clear-cutting*<br>*or* log*<br>*or* deforestation*<br>*or* fire*<br>*or* agriculture<br>*or* conversion*<br>*or* disturbance*<br>*or* degradation*<br>*or* secondary forest*<br>*or* plantation*<br>*or* fragment*	i) Measures of biodiversity at multiple sites in both primary and disturbed tropical forests<br>ii) Primary forests had little or no human disturbance<br>iii) Variance measures for biodiversity responses
Wortley et al. 2013	Web of Science (up to 2012)<br>Ecological Restoration<br>Ecological Management and Restoration	1) Restoration<br>*or* restored *and*<br>eco* *and*<br>monitor*<br>*or* success*<br>*or* evaluat*<br>*or* assess*	i) Terrestrial ecological restoration<br>ii) Outcomes for restoration post-implementation<br>iii) Active restoration including planting, weed control, fire replacement, and soil amendment<br>iv) Exluded papers that only looked at the survival of plantings<br>v) Exluded papers that only looked at where the restored site was used for production

Curran et al. 2014	Web of Science (up to 2009)<br><br>Previous reviews of:<br>Brown and Lugo (1990)<br>Houerou (2000)<br>Dunn (2004)<br>Bowen et al. (2007)<br>Liebsch et al. (2008)<br>Chazdon et al. (2009)<br>Jones and Schmitz (2009)<br>Dent and Wright (2009)<br>Rey Benayas et al. (2009)<br>Gardner et al. (2007)	1) Biodiversity<br>*or* diversity<br>*or* species diversity<br>*or* species richness<br>*and* restor*<br>*or* reclamation<br>*or* regenerat*<br>*or* regrowth<br>*or* recover*<br>*or* sucession	i) Species diversity, abundance or occurence of secondary growth and old growth habitatsii) Known age of the secondary growth habitat
Additional studies that were suggested by specialists			
